# Long-term results of early adjuvant concurrent chemoradiotherapy for high-risk, early stage uterine cervical cancer patients after radical hysterectomy

**DOI:** 10.1186/s12885-017-3299-0

**Published:** 2017-04-28

**Authors:** Sang-Won Kim, Mison Chun, Hee-Sug Ryu, Suk-Joon Chang, Tae Wook Kong, Young-Taek Oh, Seung Hee Kang

**Affiliations:** 1 Department of Radiation Oncology, Ajou University School of Medicine, 164 Worldcup-ro, Yeongtong-gu, Suwon, Gyeonggi-do 16499 Republic of Korea; 2 Department of Radiation Oncology, Konyang University College of Medicine, 158 Gwanjeodong-ro, Seo-gu, Daejeon, 35365 Republic of Korea; 3 Department of Obstetrics and Gynecology, Ajou University School of Medicine, 164 Worldcup-ro, Yeongtong-gu, Suwon, Gyeonggi-do 16499 Republic of Korea; 4 Department of Radiation Oncology, Ilsan Paik Hospital, Inje University School of Medicine, 170 Juhwa-ro, Ilsanseo-gu, Goyang, Gyeonggi-do 10380 Republic of Korea

**Keywords:** Uterine cervical neoplasm, Adjuvant chemoradiotherapy, Time to treatment, Treatment outcome, Long term adverse effects

## Abstract

**Background:**

The aim of the present study was to investigate the long-term survival outcomes and toxicities associated with our experienced early administration of adjuvant concurrent chemoradiotherapy (CCRT).

**Methods:**

Ninety-eight patients with pelvic lymph node metastasis, positive resection margin, and/or parametrial invasion who received adjuvant CCRT between 1995 and 2011 were analyzed retrospectively. The first cycle of platinum-based adjuvant chemotherapy was initiated within 2–3 weeks after surgery (median, 12 days) and continued every 4 weeks for a total of 4 cycles. Adjuvant radiotherapy was performed during the second and third cycles of chemotherapy.

**Results:**

After a median follow-up period of 119 months for survivors, 13 patients (13.3%) experienced recurrence and 11 patients died of cancer during the follow-up period. The 5-year recurrence-free survival and cancer specific survival rates were 87.6% and 90.6%, respectively. Ninety-four patients (95.9%) received ≥3 cycles of chemotherapy. Total radiation dose of ≥45 Gy was delivered in 91 patients (92.9%). Grade 3–4 hematologic and gastrointestinal toxicities developed in 37 (37.8%) and 14 (14.3%) patients during CCRT, respectively.

**Conclusion:**

The present study confirmed the long-term safety and encouraging survival outcomes of early administration of adjuvant CCRT, suggesting the benefits of early time to initiation of adjuvant treatments.

## Background

The primary treatment for International Federation of Gynecology and Obstetrics (FIGO) stage IA2–IIA uterine cervical cancer is either radical hysterectomy or external beam radiotherapy with or without concurrent platinum-based chemotherapy [1]. Although both treatment modalities have similar survival rates, radical hysterectomy is particularly preferred in some young patients because of thorough evaluation of pelvic lymph nodes, prevention of premature and avoidance of radiation-induced late toxicities. However, a considerable patient with certain adverse pathologic factors have a high possibility of recurrence despite they undergo radical surgery [2–8]. Of these, parametrial invasion, positive resection margin, and pelvic lymph node metastasis were classified as high-risk.

Adjuvant radiotherapy significantly improved 2-year recurrence-free rate in patients with intermediate risk factors compared with no further treatment [9, 10]. However, in high-risk patients, adjuvant radiotherapy alone had a limited role in locoregional control without any survival benefits [11, 12]. In 2000, the Southwest Oncology Group (SWOG), Gynecologic Oncology Group (GOG), and Radiation Therapy Oncology Group (RTOG) reported a collaborative phase III prospective randomized trial comparing the survival outcomes between adjuvant radiotherapy alone and adjuvant concurrent chemoradiotherapy (CCRT) in high-risk, early stage uterine cervical cancer patients [13]. This trial (GOG 109/SWOG 8797/RTOG 91–12) demonstrated the beneficial effects of the concomitant administration of platinum-based chemotherapy on the survival rates, as well as the disadvantage of increased treatment-related toxicities.

Over the last decade, several studies addressing various settings of adjuvant CCRT reported similar outcomes, with a 5-year recurrence free survival rate of 70–80% and a 5-year overall survival rate of 80–85% [14–20]. In all the previous studies, the first cycle of adjuvant chemotherapy was usually initiated 4–6 weeks after surgery and adjuvant radiotherapy was performed concurrently with the first cycle of adjuvant chemotherapy.

Early start of adjuvant treatments showed potential for improvement of survival outcomes in breast cancer [21], colon cancer [22] and ovarian cancer [23]. On the other hand, there have been no related literatures in cervical cancer. We previously reported the feasibility and promising results of the early administration of adjuvant CCRT, with a 5-year progression-free survival rate of 88.7% and a 5-yearoverall survival rate of 96.7% [24]. However, small sample sizes and short follow-up duration remained limitations to the study. In the present study, we reported the long-term outcomes and toxicities associated with the early administration of adjuvant CCRT for high-risk, early stage uterine cervical cancer.

## Methods

The present study was approved by the Institutional Review Board of Ajou University School of Medicine with an exemption from informed consent. We reviewed the medical records of all high-risk, early stage (FIGO IA2–IIA1) uterine cervical cancer patients who received adjuvant CCRT at our institution between 1995 and 2011. The exclusion criteria were the following: 1) class I or II radical hysterectomy, 2) small cell or neuroendocrine carcinoma, 3) para-aortic lymph node metastasis, 4) history of neoadjuvant chemotherapy and 5) initiation of adjuvant CCRT more than 3 weeks after surgery.

We performed a previously described treatment scheme [24]. In detail, after baseline staging work-ups, all patients underwent class III radical hysterectomy with bilateral pelvic lymphadenectomy and para-aortic lymph node sampling or dissection. The first cycle of adjuvant chemotherapy was initiated within 2–3 weeks after surgery. The most common treatment regimen was cisplatin (70 mg/m^2^) plus 5-fluorouracil (1000 mg/m^2^/day) (*n* = 90). Other regimens included paclitaxel (175 mg/m^2^) combined with either cisplatin or carboplatin (area under curve of 5) (*n* = 8). The combination chemotherapy regimens were administered every 4 weeks for a total of 4 cycles.

Adjuvant radiotherapy was initiated during the second course of chemotherapy. Whole-pelvic irradiation was given with a median total dose of 45 Gy in 25 fractions using a traditional four-field box technique. An additional boost of 5.4–10.8 Gy was delivered to the risky area in patients with parametrial invasion or positive lateral resection margins. Patients with positive common iliac lymph nodes received elective para-aortic irradiation with a median total dose of 44.2 Gy in 26 fractions. In patients with positive vaginal cuff resection margins, vaginal brachytherapy administered once or twice with a fraction size of 4–5 Gy after completion of external beam radiotherapy was indicated.

Acute and late treatment-related toxicities were evaluated based on the Common Terminology Criteria for Adverse Events (CTCAE) version 4.02. Adjuvant CCRT was interrupted if any grade ≥ 3 toxicities occurred. The treatment was recommenced after any signs or symptoms of severe toxicities subsided.

### Statistical analysis

The primary endpoints of the present study were the 5-year recurrence-free survival (RFS) and cervical cancer-specific survival (CSS) rates. The secondary endpoints were the 5-year locoregional recurrence free survival (LRRFS), distant metastasis-free (DMFS) and overall survival (OS) rates. All endpoints were measured from the date of radical hysterectomy and were calculated using the Kaplan–Meier method. A univariate analysis was performed with log-rank test. A multivariate analysis using factors with *p* value of <0.10 in univariate analysis was performed using the Cox proportional hazards model through backward stepwise elimination. All statistical analyses were performed using the R software version 3.2.4 (https://cran.r-project.org/). A two-sided *p* value of <0.05 was considered statistically significant.

## Results

### Patient characteristics

Between 1995 and 2001, a total of 98 patients received early administration of adjuvant CCRT within 2–3 weeks after radical hysterectomy. The patient characteristics are summarized in Table 1. The median age was 47 years (range, 26 to 71 years). Approximately two-thirds of patients (*n* = 66) had a single risk factor. The measurement of serum squamous cell carcinoma antigen before surgery was available in 96 patients, with a median value of 1.5 ng/mL (range, 0.1 to 43.9).

**Table 1:** Patient characteristics

	No. of patients (%)
Age	median, 47
Clinical FIGO stage	
IA2	1 (1.0)
IB1	71 (72.5)
IB2	24 (24.5)
IIA	2 (2.0)
Histology	
Squamous cell carcinoma	74 (75.5)
non-squamous cell carcinoma	24 (24.5)
SCC Ag	
≤ 2 ng/ml	50 (51.0)
> 2 ng/ml	46 (47.0)
unknown	2 (2.0)
Tumor size	
≤ 4 cm	50 (51.0)
> 4 cm	42 (42.9)
unknown	6 (6.1)
Deep stromal invasion	
absent	23 (23.5)
present	75 (76.5)
Lymphovascular space invasion	
absent	4 (4.1)
present	92 (93.9)
unknown	2 (2.0)
Parametrial invasion	
negative	84 (85.7)
positive	14 (14.3)
Resection margin	
negative	56 (57.1)
close (<5 mm)	9 (9.2)
positive	33 (33.7)
Pelvic lymph node metastasis	
negative	19 (19.4)
positive	79 (80.6)
LNR	
< 0.25	87 (88.8)
≥ 0.25	11 (10.2)
No. of high risk factor	
1	66 (67.3)
2	27 (27.6)
3	5 (5.1)

*FIGO* International federation of gynecology and obstetrics, *SCC Ag* squamous cell carcinoma antigen, *LNR*, lymph node ratio

### Treatment compliance

The median interval between radical hysterectomy and initiation of adjuvant chemotherapy in entire patients was 12 days (range, 8 to 21 days). The second cycle of adjuvant chemotherapy was delayed for >1 week in 6 patients owing to abscess development (*n* = 1), slow recovery from chemotherapy-related toxicities (*n* = 3), and personal reasons (*n* = 2). Ninety-four patients (95.9%) received ≥3 cycles of adjuvant chemotherapy. Chemotherapy was terminated in the remaining 4 patients before the third cycle, owing to treatment intolerability.

The median interval between surgery and the initiation of radiotherapy was 41 days (range, 28 to 70 days). Ninety-one patients (92.9%) received a total radiation dose of ≥45 Gy. The interruption of radiotherapy for >2 weeks because of treatment-related toxicities (*n* = 2) or poor compliance (*n* = 5) occurred in 7 patients.

### Survival rate

The median follow-up period was 119 months (range, 55 to 255 months) for all survivors. Recurrences occurred in 13 patients (13.3%); 4 (4.1%) of which were isolated locoregional recurrences, 4 (4.1%) were simultaneous locoregional recurrences and distant metastases, and 5 (5.1%) were distant metastases. Of these, 2 patients with lung metastasis developed 34 and 66 months after radical hysterectomy attained no evidence of disease after salvage metastatectomy.

**Fig. 1:**
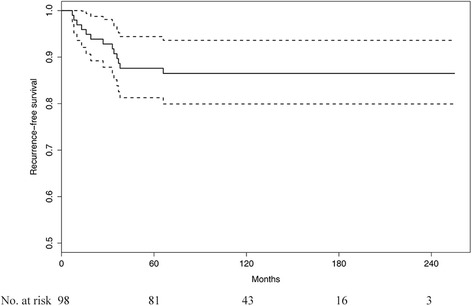
Kaplan-Meier curve of recurrence-free survival for all patients (*dotted line*, 95% confidence interval)

The 5-year LRRFS, DMFS and RFS rates were 91.5% (95% confidence interval [CI], 86.0–97.3%), 90.6% (95% CI, 84.9–96.6%), and 87.6% (95% CI, 81.3–94.4%), respectively (Fig. 1). In univariate analysis, the number of positive pelvic lymph nodes ≥3 (*p* = 0.021), and lymph node ratio ≥ 0.25 (*p* = 0.018) were significantly associated with poor RFS. Multivariate analysis revealed the number of positive lymph nodes ≥3 as a significant prognostic factor (hazard ratio [HR], 3.179; 95% CI, 1.066–9.477; *p* = 0.038) (Table 2).

**Table 2:** Univariate and multivariate analyses for RFS and CSS

	RFS					CSS				
variables	5 yr. rate	*p* value	HR	95% CI	*p* value	5 yr. rate	*p* value	HR	95% CI	*p* value
size >4 cm		0.304					0.073	2.781	0.710–10.990	0.142
no	92.0					93.9				
yes	83.1					87.9				
SCC Ag		0.611					0.904			
≤ 2 ng/mL	88.0					89.9				
> 2 ng/mL	86.9					91.2				
DSI		0.985					0.778			
absent	86.4					90.7				
present	87.9					90.6				
LVSI		0.671					0.720			
absent	100.0					100.0				
present	86.9					90.1				
PM invasion		0.145					0.196			
absent	85.7					89.2				
present	100.0					100.0				
RM		0.052	2.868	0.881–9.333	0.080		0.132			
clear	92.8					92.7				
close or positive	81.0					88.0				
LN metastasis		0.664					0.932			
absent	88.8					88.2				
present	87.3					91.1				
No. of positive LN		0.021	3.179	1.066–9.477	0.038		0.005	4.004	1.130–14.195	0.032
0–2	92.8					94.5				
≥ 3	74.1					81.5				
LNR		0.018	2.330	0.611–8.879	0.215		0.011	1.740	0.391–7.740	0.467
< 0.25	90.7					91.8				
≥ 0.25	63.6					81.8				
No. of risk factors		0.299					0.345			
1	90.8					90.7				
2–3	81.3					90.6				

*RFS* recurrence free survival rate, *CSS* cancer specific survival rate, *HR* hazard ratio, *CI* confidence interval, *SCC Ag* squamous cell carcinoma antigen, *DSI* deep stromal invasion, *LVSI* lymphovascular space invasion, *PM* parametrium, *RM* resection margin, *LN* lymph node, *LNR* lymph node ratio

**Fig. 2:**
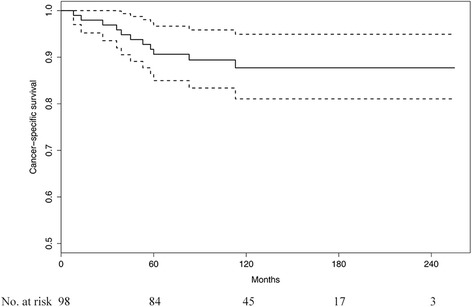
Kaplan-Meier curve of cancer-specific survival for all patients (*dotted line*, 95% confidence interval)

At the last follow-up, 11 patients died of cancer progression and 4 died of intercurrent causes. The 5-year CSS and OS were 90.6% (95% CI, 85.0–96.7%) and 88.7% (95% CI, 82.7–95.2%), respectively (Fig. 2). Univariate analysis identified the number of positive pelvic lymph nodes ≥3 (*p* = 0.005) and lymph node ratio ≥ 0.25 (*p* = 0.011) as the significant risk factors for CSS. In multivariate analysis, the number of positive pelvic lymph nodes ≥3 (HR, 4.004; 95% CI, 1.130–14.195; *p* = 0.032) was the only prognostic factor for CSS (Table 2).

### Toxicities

Hematologic toxicities of CTCAE grade 3–4 occurred in 37 patients (37.8%; Table 3). Of these, febrile neutropenia developed in 2 patients (2.0%) and they recovered after antibiotic therapy. Fourteen patients (14.3%) developed grade ≥ 3 gastrointestinal toxicities during adjuvant CCRT. Of these, 3 patients (3.1%) developed grade 4 small bowel obstruction and they recovered fully after successful surgical intervention. One patient died of panperitonitis due to small bowel perforation 7 years after the completion of adjuvant CCRT.

**Table 3:** Grade 3 or more Treatment-related toxicities

variables	Percent
Hematologic	
leukopenia	36.5
neutropenia	11.5
anemia	8.7
thrombocytopenia	1.0
Gastrointestinal	
anorexia	1.9
nausea/vomiting	1.9
diarrhea	6.7
proctitis	1.0
small bowel obstruction	4.8
Lymphedema	1.0

## Discussion

Consistent with the results of our previous study [24], the present study demonstrated the safety and encouraging treatment outcomes of the early administration of adjuvant CCRT.

In the current clinical practice, adjuvant CCRT for high-risk, early stage uterine cervical cancer is usually allowed to initiate 4–6 weeks after radical hysterectomy, providing time for wound healing. Therefore, it can be assumed that early initiation of adjuvant CCRT within 2–3 weeks from radical surgery potentially causes delayed wound healing. However, Kolb et al. reported that early initiation of adjuvant chemotherapy was not related to any increase in wound complications after cytoreductive surgery for ovarian cancer [25]. In the present study, the incidence of postoperative morbidities was similar to that of previous studies, confirming the safety of early administration of adjuvant CCRT [13–20]. The timely initiation of the second course of adjuvant chemotherapy and radiotherapy in the majority of patients (95.8%) supported this finding.

Early administration of adjuvant CCRT has another possible concern of increasing treatment-related toxicities because of inadequate recovery from radical surgery. However, the incidence rates of grade 3–4 treatment-related toxicities in the present study were comparable to those reported by the previous studies [13–20]. We did not observe any late toxicities related to early administration of adjuvant CCRT. Thus, the present study reconfirmed the safety of the early administration of adjuvant CCRT with long-term follow-up.

In the present study, the 5-year RFS and CSS rates were 87.6% and 90.6%, respectively. An indirect comparison with previous studies showed that the survival outcomes in the present study appeared to be more favorable [13–20]. We used chemotherapeutic regimen based on contemporary guidelines and conventional radiotherapy. The major discrimination between the present study and previous studies was the time to initiation of adjuvant treatments. Therefore, early administration of adjuvant CCRT might have potential for improvement of adjuvant treatment outcomes.

The benefit of early administration of adjuvant CCRT could be explained by the properties of chemotherapeutic agents. In a preclinical experiment, Gunduz et al. observed that the surgical removal of tumor converted dormant tumor cells into rapidly proliferating ones [26]. In a subsequent study, they demonstrated that long interval between surgical removal of primary tumor and administration of chemotherapy attenuated the cytotoxic effects of the drug, manifested by a decrease in the proliferative index of tumor cells and a gradual increase in the total tumor volume over time [27]. Considering the high sensitivity of the chemotherapeutic agents to rapidly proliferating cells and lower tumor burden, the immediate administration of adjuvant treatments after surgery as possible could maximize their efficacy.

In addition, early administration of adjuvant CCRT contributed to improved survival rates in a different way. The major concern of adjuvant CCRT for high-risk, early stage uterine cervical cancer was poor compliance due to synergistic effects of each treatment-related toxicities. Approximately a quarter of patients ceased further chemotherapy before the third cycle of chemotherapy in the GOG 109/SWOG 8797/RTOG 91–12 trial. Furthermore, patients who discontinued further chemotherapy after the completion of radiotherapy had significantly lower survival rates than those who received ≥3 cycles of chemotherapy (*p* = 0.03 for both progression-free and overall survival), demonstrating the favorable effect of a higher number of chemotherapy courses. Early administration of adjuvant CCRT provided a countermeasure through the delivery of adjuvant radiotherapy at the second cycle of adjuvant chemotherapy without deferring the time to initiation of irradiation (median, 41 days). Therefore, ≥3 cycles of chemotherapy were guaranteed in the majority of the patients even though further chemotherapy was not administered following the completion of radiotherapy at the patients’ discretion. As mentioned above, the majority of patients (95.9%) in the present study received ≥3 cycles of chemotherapy and the survival outcomes were comparable with those who received ≥3 cycles of chemotherapy in the GOG 109/SWOG 8797/RTOG 91–12 trial. Based on this finding, the higher number of chemotherapy courses owing to early administration of adjuvant CCRT might contribute to the improvement of adjuvant treatment outcomes.

The present study had several limitations. First, the sample size was not large enough to draw definite conclusions. Second, the present study might have selection bias because of its retrospective nature. Finally, the details of adjuvant treatment were heterogeneous in the minority of patients. However, given the similar survival rates among different chemotherapeutic regimen in previous studies and few evidence that demonstrated survival benefit by vaginal brachytherapy in the adjuvant setting of cervical cancer, it is unlikely that heterogeneities in the treatment approach influenced treatment outcomes.

## Conclusion

In summary, the present study demonstrated encouraging adjuvant treatment outcomes in high-risk cervical cancer patients, possibly owing to early time to initiation of adjuvant CCRT. The treatment compliance and toxicities were also at the comparable levels. Therefore, the present study might suggest a direction for further improvement of adjuvant treatment for high-risk, early stage uterine cervical cancer. Whether this treatment strategy is effective in only selected patients, further studies with a prospective design are warranted.

## Abbreviations

CCRT: Concurrent chemoradiotherapy
CI: Confidence interval
CSS: Cancer-specific survival
CTCAE: Common Terminology Criteria for Adverse Events
DMFS: Distant metastasis-free survival
FIGO: International Federation of Gynecology and Obstetrics
GOG: Gynecologic Oncology Group
HR: Hazard ratio
LRRFS: Loco-regional recurrence free survival
OS: Overall survival
RFS: Recurrence-free survival
RTOG: Radiation Therapy Oncology Group
SWOG: Southwest Oncology Group
